# TPS1 drug design for rice blast disease in magnaporthe oryzae

**DOI:** 10.1186/2193-1801-3-18

**Published:** 2014-01-10

**Authors:** Yangkui Xue, Guanghou Shui, Markus R Wenk

**Affiliations:** Department of Biochemistry, Yong Loo Lin School of Medicine, National University of Singapore, 28 Medical Drive, Singapore, 117456 Singapore; Institute of Genetics and Developmental Biology, Chinese Academy of Sciences, No.1 West Beichen Road, Chaoyang District, Beijing, 100101 China

**Keywords:** *M. oryzae*, Rice blast disease, Trehalose-6-phosphate synthase 1, Tps1, Fungus, Molecular dynamics simulation

## Abstract

*Magnaporthe oryzae* (*M. oryzae*) is a fungal pathogen and the causal agent of rice blast disease. Previous lipidomics analysis of *M. oryzae* demonstrated that trehalose, a carbohydrate common to various fungi and algae, is thought to be involved in the possible conversion of glycogen into triacylglycerides for energy, an important step in the pathogenesis of *M. oryzae*. A key enzyme responsible for trehalose synthesis is trehalose-6-phosphate synthase 1 (Tps1). Therefore, we modeled the structure of Tps1 and sought to screen a chemical database *in silico* for possible inhibitors of the enzyme. Based on homologous alignment and sequence analysis, we first modeled the structure of Tps1 to determine the potential active site of the enzyme and its conformation. Using this model, we then undertook a docking study to determine the potential interaction that would manifest between Tsp1 and potential chemical inhibitors. Of the 400,000 chemicals screened in the Molecular Libraries Small Molecule Repository, we identified 45 potential candidates. The best candidate (Compound 24789937) was chosen and subjected to various structural optimization techniques to improve the suitability of the potential chemical inhibitors at the docking site of Tps1. From these modified versions of Compound 24789937, one lead compound (Lead 25) was shown to have the best binding affinity to Tps1 and good water solubility as compared with the ideal template compound and the other 44 potential candidates. Molecular dynamics simulation further confirmed the strength of the Tps1–Lead 25 complex and indicated the potential for Lead 25 to be used as an inhibitor of Tps1 in the control of *M. oryzae*–mediated rice blast disease.

**Electronic supplementary material:**

The online version of this article (doi:10.1186/2193-1801-3-18) contains supplementary material, which is available to authorized users.

## Background

Trehalose (α-D-glucopyranosyl-α-D-glucopyranoside) is a natural alpha-linked disaccharide common to various fungi, algae and some invertebrate phyla (Paiva and Panek 1996). It has important functions in energy and carbon storage, it acts as a signaling molecule for growth in yeast and fungi (Silva et al., 2005), and it offers a protective role for cellular membranes and proteins under various stressors such as desiccation, dehydration, heat, cold, and oxidation (Leslie et al., 1995; Santos and Da Costa 2002; Elbein et al., 2003; Silva et al., 2003). In light of these important functions in fungi, there have been numerous attempts to develop anti-fungal reagents that interfere with trehalose metabolism; for instance, 3,3P-diketotrehalose (3,3PdkT) is a novel trehalose derivative that has an inhibitory effect toward *Bombyx mori* trehalase (Sode et al., 2001), the hydrolase that catalyzes the conversion of trehalose to glucose.

*Magnaporthe oryzae* (*M. oryzae*) is a hemibiotrophic fungal pathogen that causes rice blast disease, the most severe disease affecting cultivated rice (Talbot 2003). Rice blast disease currently represents a serious threat to global rice production, and various efforts are being made to inhibit the pathogenic effects of *M. oryzae*. In order to breach the plant cuticle and cause infection, fungi such as *M. oryzae* create an infection structure called an appressorium, which accumulates high molar concentrations of glycerol and generates hydrostatic turgor by drawing water into the cell. Triacylglycerides, or TAGs, are a major source of glycerol used to generate turgor pressure for *M. oryzae* penetration into the host (Thines et al., 2000), the concentration of which significantly increases during the early stages of pathogenesis. On the other hand, glycogen is metabolized before the onset of turgor pressure (Howard and Valent, 1996; Thines et al., 2000). Though it was suspected glycogen could be used for glycerol production, no mechanism has been suggested yet.

We have previously proposed a possible model for turgor pressure production (Yangkui Xue, et. al., *manuscript in preparation*). In this model, we propose that trehalose synthesis might play a role in the successful conversion of glycogen into TAGs, for two reasons. (1) During the first 2 hours of conidia germination, trehalose concentrations increase but are soon completely degraded, whereas trehalase activity remains high during the entire period of germination (Foster et al., 2003). The co-occurance of glycogen breakdown, the synthesis of TAGs and trehalose during the early stage of pathogensis may be suggestive of a link between sugar metabolism (i.e., that of trehalose) and turgor production. (2) A deeper look into the function of trehalose reveals its regulatory role in glycolysis and gluconeogenesis (Foster et al., 2003; Thevelein and Hohmann 1995). Therefore, it is plausible to suggest that the synthesis and (rapid) breakdown of trehalose ensures the successful conversion of glycogen into TAGs. Hence, it may be possible to interfere with turgor production in *M. oryzae* by inhibiting trehalose synthesis.

Trehalose synthesis requires trehalose-6-phosphate synthase 1 (Tps1), together with trehalose-6-phosphate phosphatase (T6pp) (Gibson et al., 2002). In *M. oryzae*, Tps1 comprises 529 amino acids (GenBank: AAN46744.1), and previous studies have indicated that there are four amino acids within the active site that are important for trehalose production, sporulation and pathogenicity (Wilson et al., 2007). Furthermore, Tps1 is responsible for the production of trehalose and the utilization of nitrogen, as well as the regulation of several NADPH-dependent transcriptional co-repressors, namely Nmr1–3, all of which bind NADPH (Wilson et al., 2010). These results suggest that Tps1 could be a possible target for the production of anti-fungal inhibitors.

Thus, the aim of this study was to screen for potential inhibitors of Tsp1 as a means to inhibit trehalose synthesis and/or action in *M. oryzae* using structure modeling experiments *in silico*.

## Results and discussion

### Structure modeling by modeller

**Figure 1:**
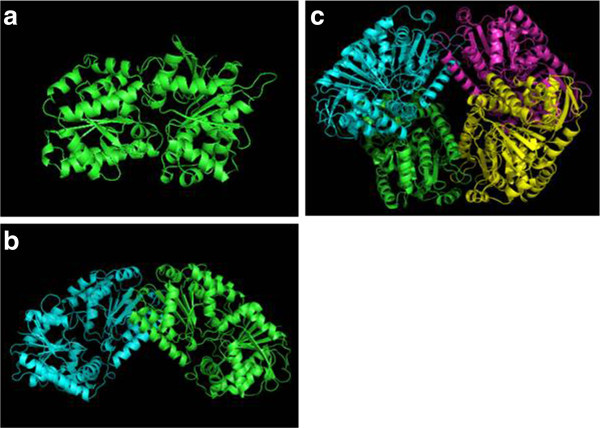
**Homologous modeling of Tps1 based on 2wtx, 1gz5, 1uqu, and 1 uqt. (a)** Tps1 monomer; **(b)** Tps1 dimer; **(c)** Tps1 tetramer.

**Figure 2:**
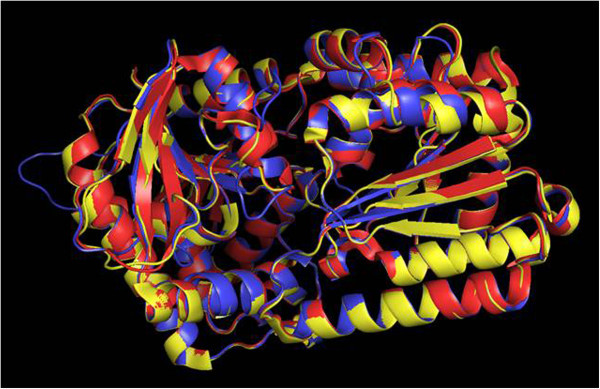
**The aligned monomer of tps1 (blue), 1gz5 (red) and 2wtx (yellow) by Pymol (The PyMOL Molecular Graphics System, Version 1.5.0.4 Schrödinger, LLC0.**

The protein sequence of Tps1 was compared against the Protein Data Bank (PDB) using the BLAST alignment tool (NLM, Bethesda, MD), and several homologous proteins—2wtx (Errey et al., 2010), 1gz5 (Gibson et al., 2002) and 1uqt/1uqu (Gibson et al., 2004)—were identified and subsequently used as templates for structure modeling by Modeller (Šali and Blundell 1993). Multiple sequence alignment of the homologs with Tps1 revealed a possible signaling peptide at the N-terminal of Tps1 (Additional file 1: Figure S1), which was subsequently removed during the actual sequence alignment (Additional file 2: Figure S2) and structure modeling. Because the homologs of Tps1 may adopt different conformations (i.e., monomer, dimers and tetramers) (Gibson et al., 2002), the potential different conformational structures of Tps1 were also modeled (Figure 1). The monomers of Tps1, 1gz5 and 2wtx were aligned and compared using Pymol (The PyMOL Molecular Graphics System, Version 1.5.0.4 Schrödinger, LLC) (Figure 2). The root mean square deviations (RMSD) of the alignment were between 0.11 and 0.29, indicating a similar structure between Tsp1 and the Templates. A further look into the structure also revealed the presence of the central core of seven parallel strands and six helices at the N-terminus and a Rossmann-fold at the C-terminus; these structures were also found in 1gz5 (Gibson et al., 2002).

The quality of the model by produced using Modeller was compared with that made by auto-modeling at the Phyre server (Kelley and Sternberg 2009). Both models were assessed using Anolea (Hynes et al., 2008), Errat (Shen and Burger, 2009) and verify3D (Shui et al., 2011; Luthy et al., 1992), and the model by Modeller was found to be of a better quality (data not shown). It was therefore concluded that the Tps1 model by Modeller was suitable for our subsequent docking study.

### Screening of MLSMR for inhibitors

**Figure 3:**
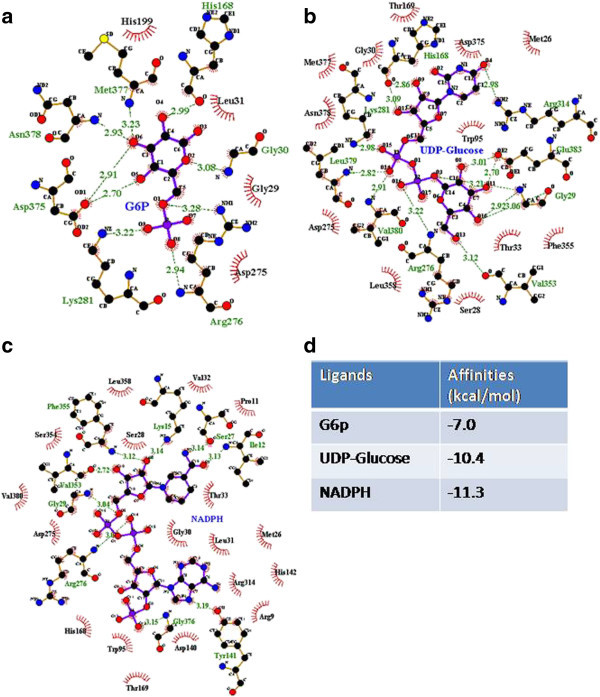
**Validation of the performance of AutoDock Vina software (Trott and Olson,** 2010 **).** The binding conformations of **(a)** glucose-6-phosphate (G6P), **(b)** uridine-diphosphate-glucuse (UDP-Glucose) **(b)** and **(c)** NADPH when docked onto Tps1. **(d)** Binding affinities of each ligand with Tsp1.

The active site of Tps1 was identified based on the binding conformations of 2wtx, 1gz5 and 1uqt/1uqu with their corresponding ligands using Pymol. To evaluate the performance of AutoDock Vina (Trott and Olson 2010) to search for chemicals with high binding affinities for Tps1, glucose-6-phosphate (G6p), uridine diphosphate glucose (UDP-Glucose) and nicotinamide adenine dinucleotide phosphate-oxidase (NADPH) were docked onto Tps1 (Figure 3a-3c), and their binding affinities were recorded (Figure 3d). Overall, the measured binding affinities to Tps1 were smaller than those reported in earlier work (Wilson et al., 2010), possibly due to differences in the docking software used in the two studies. Despite this, our values and those of Wilson et al. still showed a similar trend: G6p and UDP-Glucose bind to Tps1 with similarly affinities, whereas NADPH binds with higher affinity.

The binding conformations were analyzed, and we identified that G6p, UDP-Glucose and NADPH formed hydrogen bonds with Gly29, His168, Arg276, Lys281 and Val353 more easily than with other resides. In addition, several residues (Met26, Ser28, Gly30, Leu31, Thr33, Trp95, Thr169, and Asp275) showed higher contributions to hydrophobic interactions than other residues. All of these findings were consistent with previous results (Wilson et al., 2007).

Using this data, we then tested the accuracy of AutoDock Vina for its use in screening for potential interacting compounds. Indeed, we found that AutoDock Vina was able to predict a similar binding interaction with a fast screening speed (about 5–10 second/ligand) at the default level of exhaustiveness. Thus, AutoDock Vina was chosen as a screening tool to identify potential compounds in the Molecular Libraries Small Molecule Repository (MLSMR) database.

**Figure 4:**
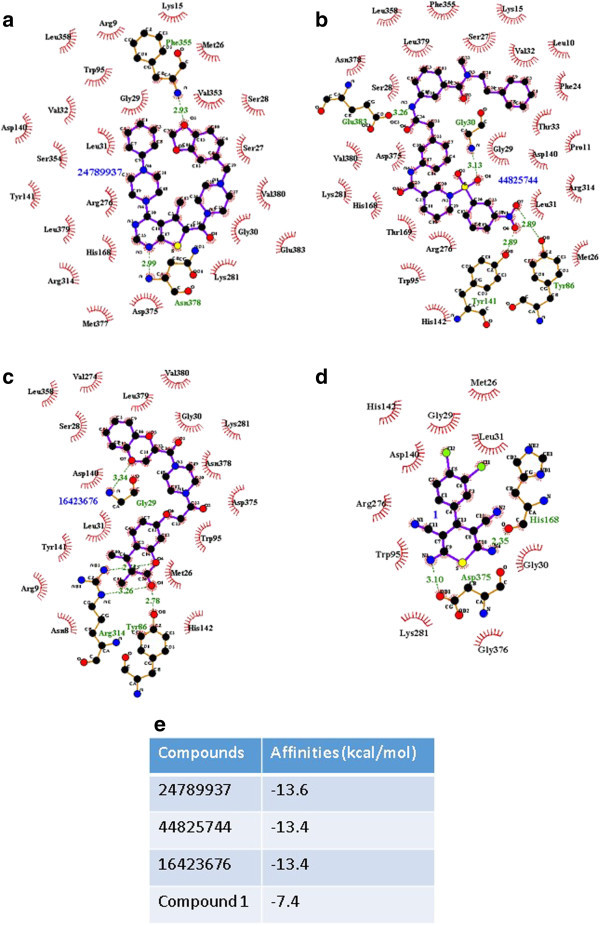
**Binding conformations of the best three compounds from the Molecular Libraries Small Molecule Repository (MLSMR), when docked onto Tps1. (a)** 24789937, **(b)** 44825744, and **(c)** 16423676, and the binding conformation of Compound 1 **(d)** (Kern et al., 2012). **(e)** Binding affinity measurements.

All of the approximately 400,000 compounds in MLSMR were screened by AutoDock Vina in terms of their binding affinities for Tps1. Of these, 45 compounds with the highest binding affinities were selected for further analysis (Additional file 3: Table S1). The binding conformations of the best three compounds—24789937, 44825744 and 16423676— are shown (Figure 4a-4c) and the binding affinities are also indicated (Figure 4e). (All of the compound conformations are provided in Additional file 4: Figure S3.)

In our analysis of the 45 compounds, we found several similarities among the compounds in terms of the formation of H-bonding and hydrophobic bonding with Tps1. Indeed, compounds with a high affinity for Tps1 formed hydrogen bonds with Gly29, Gly30, Leu31, Tyr141, Arg276, Lys281, Arg314, Asn378 and Leu379. Other residues, such as Gly31, Tyr86, Arg277 and Met377, may also contribute to binding, but the interactions with these residues were observed much less frequently among the 45 compounds. In terms of hydrophobic interactions, residues such as Ser28, Gly29, Gly30, Leu31, Trp95, Asp140, Lys281, Leu358, Asp375 and Val380 were found to interact with the 45 compounds more frequently as compared with other residues, such as Thr33, His168, Arg276, Phe355 and Leu379 that were less involved in the interaction. These results correlated well with a previous knock-out study that showed that Trp95 and Asp140 were part of the active site (Wilson et al., 2007). In addition, the screen identified that other residues, such as Ser28 and Gly29, would be good potential targets for drug design in future, because of their frequent involvement in hydrophobic interactions. The screening results also identified the key residues that are likely to be crucial in the Tps1-ligand interaction. Finally, the compounds identified to have high binding affinities with Tps1 may also provide insight into the desired features of candidate compounds for inhibiting the activity of Tps1.

As a comparison, we tested the binding affinity of a compound identified from a previous high-throughput screening for potent inhibitors of the Tps from the cat flea *Ctenocephalides felis* and *Drosophila melanogaster* (Kern et al., 2012) with *M. oryzae* Tps1 (Figure 4d and 4e). Surprisingly, the compound (referred to as ‘Compound 1’) did not bind well to Tps1, which may be due to structural variations between the different Tps enzymes; indeed, Compound 1 is a relatively smaller molecule as compared with the compounds identified by AutoDock Vina, with much potential for further modification.

### Optimization of lead 25

**Figure 5:**
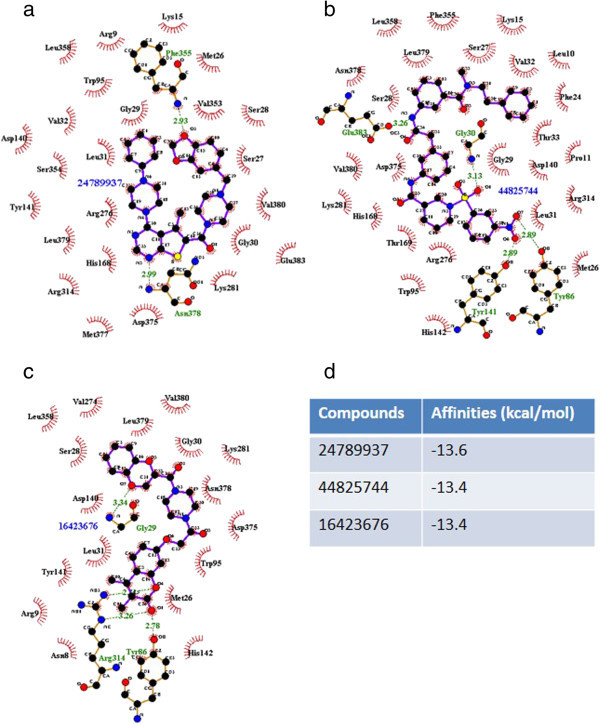
**The binding confirmations of the best 3 compounds when docked into Tps1. (a)** 24789937, **(b)** 44825744, **(c)** 16423676, **(d)** the corresponding affinities of the compounds.

Of the 45 compounds, we finally selected Compound 24789937 as a template for the further optimization of candidate compounds because it had the highest binding affinity and because it formed no H-bond interactions with the protein; this would thus allow us to perform various optimizations to modify the compound. Based on the predicted logP value, however, Compound 24789937 was shown to have very low water solubility (Figure 5e). Water solubility of a compound can be improved by the introduction of charged groups into the chemical structure, and it is desirable if these charged groups also form H-bonds with the residues at the active site. Based on these two guidelines, 26 structural modifications were made to Compound 24789937 and the various modified forms of the molecule were then re-docked onto Tps1 (Additional file 5: Figure S4). We found, however, that most of these modified chemicals could not bind to Tps1 as strongly as Compound 24789937, with the exception of one of the compounds, Lead 25, for which a binding affinity of -13.8 kcal/mol was measured. Our analysis showed that the introduced ketone group on both Lead25 (Figure 5a and 5b) and Compound 24789937 (Figure 5c) increased the negative electrostatic potential around the central portion of both molecules. On Lead 25, this ketone group formed H-bonds with Ser7 and Arg9 of Tps1 (Figure 5d), and also greatly increased the compound’s water solubility (Figure 5e). Thus, the modifications made to Compound 24789937 led to the successful identification of Lead 25, a compound that showed high affinity binding with Tps1 and good water solubility.

### Molecular dynamics by GROMACS

**Figure 6:**
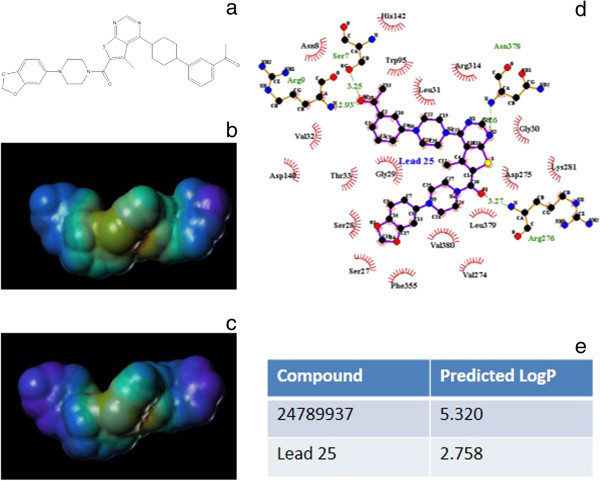
**Lead 25 was shown to interatct strongly with Tps1. (a)** the RMSD of backbones of Tps1, Tps1-G6p complex, Tps1-NADPH complex, Tps1-UDP-Glucose complex and Tps1-Lead 25 complex during 10 ns of simulation; **(b)** the number of H-bonds of Tps1-G6p complex, Tps1-NADPH complex, Tps1-UDP_Glucose complex and Tps1-Lead 25 complex during 10 ns of simulation; **(c)** the energies between the ligands and Tps1, with the unit being kJ/mol; **(d)** the 2D binding confirmation of Tps1-Lead 25 plotted by Ligplot, **(e)** the predicted logP values of Lead 25 and Compound 24789937.

From the analysis of the backbone structures of each chemical (Figure 6a), it was obvious that all of the ligands (G6p, UDP-Glucose, NADPH and Lead 25) could form stable complexes with Tps1 after 2 ns, since the RMSD values were found to be rather constant (about 0.3 nm) after this time point. Yet, significant H-bond formation was observed between Tps1 and Lead 25 during these first 2 ns (Figure 6b), which was not the case for the Tps1–UDP-Glucose complex. In addition to having the highest binding affinity among the modified compounds, not surprisingly, the Tsp1–Lead 25 interaction also had high energy recordings (Figure 6c), although, for two of the three energy measurements, Tsp1–NADPH was slightly better or the same as Tsp1–Lead 25. Despite this, the molecular dynamics simulation data collectively indicate that Lead 25 is likely to interact more strongly with Tps1 than the other compounds and therefore offers a good potential candidate inhibitor of the enzyme.

## Conclusion

Tps1 plays a significant role in the pathogenesis of *M. oryzae*. This study sought to identify suitable inhibitors from the MLSMR database using modeling experiments of the 3D structure of Tps1. From a selection of 45 candidate inhibitors, we identified Lead 25 as a possible Tps1 inhibitor, with a strong binding affinity toward Tps1 and good water solubility. Molecular dynamic simulation further verified the potential validity of Lead 25 as a potential inhibitor of Tsp1 activity. Future research will focus on the synthesis of Lead 25 and its potential efficacy as an inhibitor of Tsp1in rice blast control.

## Methods

### Homologous modeling of Tps1’s structure

The 2D sequence of Tps1 was used to BLAST against the proteins in the PDB, and a few homologous proteins were identified and subsequently used as the templates for structure modeling by Modeller (Šali and Blundell 1993). The PDB identifiers—2wtx, 1gz5, 1uqu and 1uqt—are described in the Results section. During the multiple sequence alignment, a possible signal peptide of Tps1, comprising 13 amino acids, was also discovered and excluded during the actual structure modeling.

Based on the conformations of the homologous proteins, Tps1 may exist as a monomer, dimer or tetramer. Thus, for each potential conformation, four structures were modeled using the loop method of Modeller, and all of the qualities of each structure were assessed using Anolea (Hynes et al., 2008), Errat (Shen and Burger 2009) and verify3D (Shui et al., 2011; Luthy et al., 1992). The structures with the best qualities overall were then selected as the final models.

The 1D sequence of Tps1 was also submitted to the Phyre server (Kelley and Sternberg 2009) for automatic modeling, and the quality of this model was compared with that of the model developed using Modeller.

### Screening of MLSMR by AutoDock Vina and analysis of docking results

All compounds in MLSMR were screened using AutoDock Vina (Trott and Olson 2010) to search for chemicals with high binding affinities for Tps1. The size of the grid box was set as: center_x = 1, center_y = 1.357, center_z = 50; size_x = 18, size_y = 20, size_z = 20. The exhaustiveness level was set to 8. Compounds with highest binding affinities were then analyzed and considered as possible templates for further optimization.

When analyzing ligand-protein interactions, Ligplot (Wallace et al., 1995) was used to plot the 2D schematic diagrams of the hydrophobic interaction and H-bonding. The lipophilicity of a compound was calculated using virtual logP (Gaillard et al., 1994). The virtual logP was preferred because the 3D conformation of the compound would be taken into consideration during the calculation.

### Lead optimization

Compounds with highest binding affinities were selected and their structures analyzed in terms of suitability for further modification, with H-bonding being one criteria. Finally, Compound 24789937 was chosen as the template, and 26 modifications were performed using ChemDraw® 8.0 and Chem3D® 8.0 (CambridgeSoft, Cambridge, MA). Each of the 26 compounds was docked into Tps1 to assess the binding affinity. SYBYL-*X*2.0 (Tripos, St. Louis, MO) was used to plot the electrostatic potential of the compounds.

### Molecular dynamics by GROMACS

Molecular dynamics simulation of the complex was carried out with the GROMACS 4.5.4 package using the GROMOS96 43a1 force field (Berendsen et al., 1995; Lindahl et al., 2001). The docking conformations with the highest binding affinities generated by AutoDock Vina for G6P, NADPH, UDP-Glucose and Lead 25 were taken as initial conformations for molecular dynamics simulation. At the same time, Tps1 in the absence of ligand was also simulated as a negative control for the study of the ligand-protein interaction. The topology parameters of Tps1 were created using the GROMACS program, while those of the ligands were built by the Dundee PRODRG server (Schuttelkopf and Van Aalten 2004). Each of the complexes was immersed in a dodecahedron box of simple point charge (SPC) water molecules (Van Gunsteren et al., 1996). The solvated system was neutralized by adding 9 Na^+^ ions. To release conflicting contacts, energy minimization was performed using the steepest descent method of 50,000 steps. Two steps were for molecular dynamics simulation studies: equilibration phase and production phase. For equilibration, the system was subjected to the position-restrained dynamics simulation (NVT and NPT) at 300 K for 100 picoseconds (ps). Finally, the full system was subjected to MD production run at 300 K temperature and 1 bar pressure for 10 ns. The atom coordinates were recorded at every 2 ps during the MD simulation.

The simulation results were analyzed using the backbone RMSD values of Tps1, the H-bonding between Tps1 and the ligands, as well as some energies, such as the Lennard Jones–Short Range (LJ–SR), the Lennard Jones–Long Range (LJ–LR) and the Coulombic potential–Short Range (Coul–SR) energies from 0 ps to 3000 ps were analyzed. A movie was also created based on the trajectories during simulation.

## Electronic supplementary material

Additional file 1: Figure S1: Multiple sequence alignment of translated sequence of Tps1, the translated (GI: 191173009) and experimentally identified sequences of 2wtx. It was used to identify the possible signaling peptide of Tps1 and to prepare a truncated version of Tps1 sequence for structural modelling. (JPEG 55 KB)

Additional file 2: Figure S2: Multiple sequence alignment of the truncated Tps1, 2wtx, 1gz5 and 1uqt. (JPEG 112 KB)

Additional file 3: Table S1: The top 45 compounds with the lowest-energy binding confirmations are listed here, with their binding energies specified as well, and the unit for affinity is kcal/mol. (JPEG 94 KB)

Additional file 4: Figure S3: The top 45 compounds with the strongest binding confirmations are listed here, and the 2D interactions with Tps1 were also presented, which were prepared by Ligplot. (ZIP 920 KB)

Additional file 5: Figure S4: the 2D structures of the 26 modified compounds based on Compound 24789937 as the template, with the corresponding affinities were also included in the brackets. (ZIP 140 KB)

## Abbreviations

MLSMR: Molecular Libraries Small Molecule Repository
G6p: Glucose-6-phosphate
UDP-Glucose: Uridine diphosphate glucose
NADPH: Nicotinamide adenine dinucleotide phosphate-oxidase
RMSD: Root mean square deviation.
