# Supplementary figures and images for: TPS1 drug design for rice blast disease in magnaporthe oryzae
Source: Springerplus. 2014 Jan 10;3:18. doi: 10.1186/2193-1801-3-18 (PMC3901853; doi:10.1186/2193-1801-3-18)

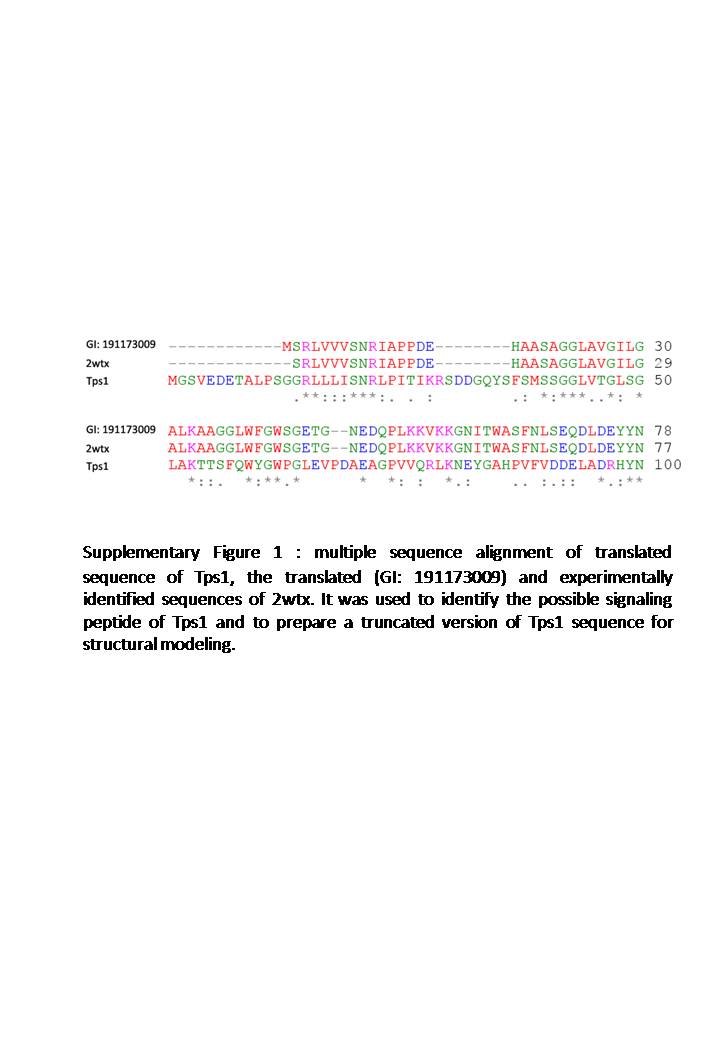

Supplement: Supplementary file 1 — Additional file 1: Figure S1: Multiple sequence alignment of translated sequence of Tps1, the translated (GI: 191173009) and experimentally identified sequences of 2wtx. It was used to identify the possible signaling peptide of Tps1 and to prepare a truncated version of Tps1 sequence for structural modelling. (JPEG 55 KB) [file 40064_2013_782_MOESM1_ESM.jpeg]

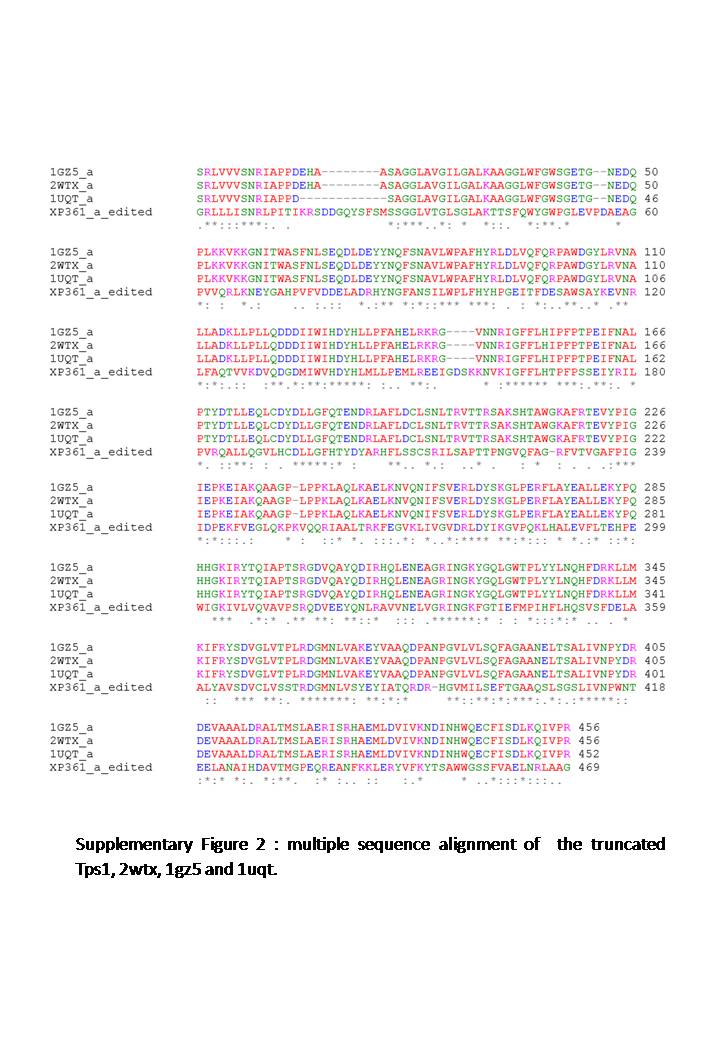

Supplement: Supplementary file 2 — Additional file 2: Figure S2: Multiple sequence alignment of the truncated Tps1, 2wtx, 1gz5 and 1uqt. (JPEG 112 KB) [file 40064_2013_782_MOESM2_ESM.jpeg]

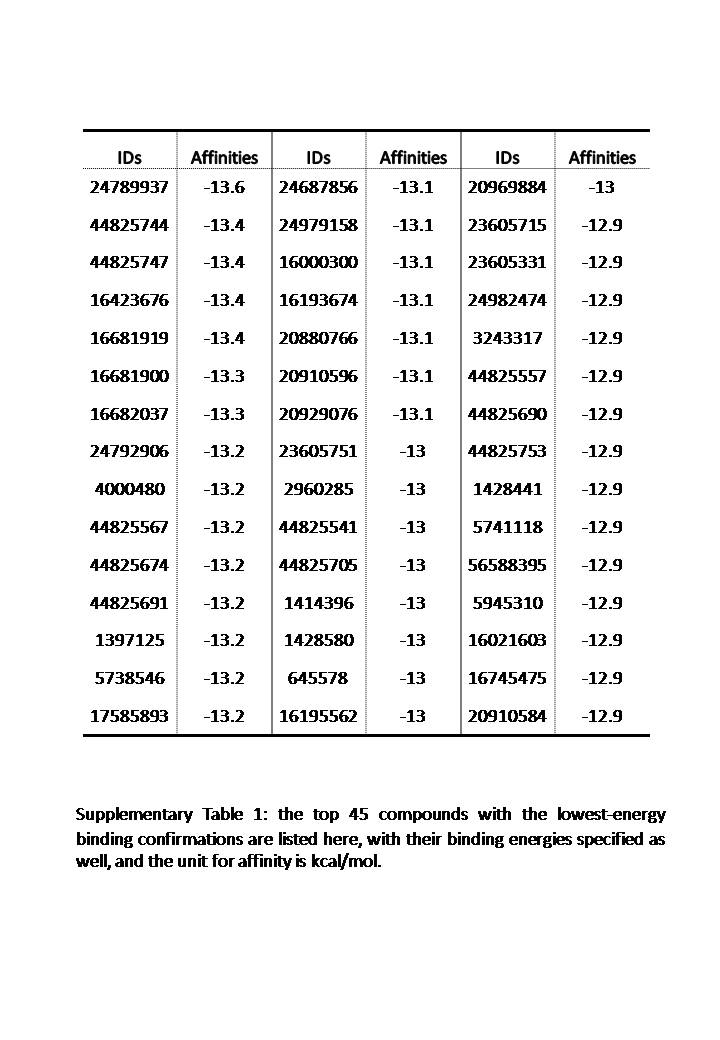

Supplement: Supplementary file 3 — Additional file 3: Table S1: The top 45 compounds with the lowest-energy binding confirmations are listed here, with their binding energies specified as well, and the unit for affinity is kcal/mol. (JPEG 94 KB) [file 40064_2013_782_MOESM3_ESM.jpeg]

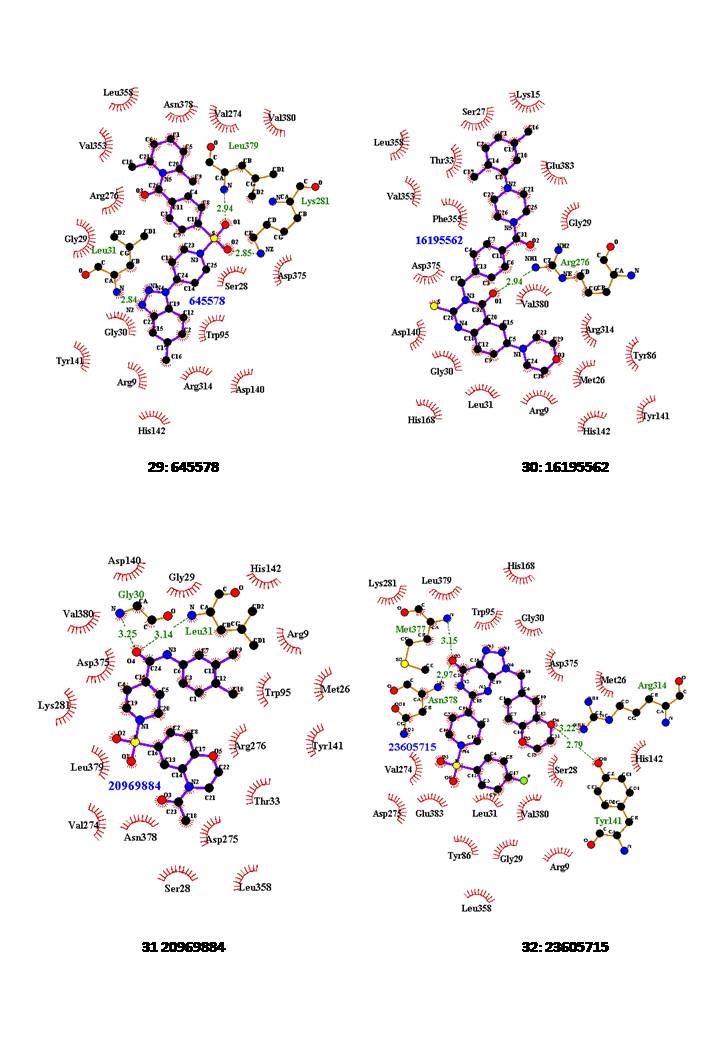

Supplement: Supplementary file 4 — Additional file 4: Figure S3: The top 45 compounds with the strongest binding confirmations are listed here, and the 2D interactions with Tps1 were also presented, which were prepared by Ligplot. (ZIP 920 KB) [file 40064_2013_782_MOESM4_ESM.zip › 8735656271096913_add3/8735656271096913_add10.jpeg]

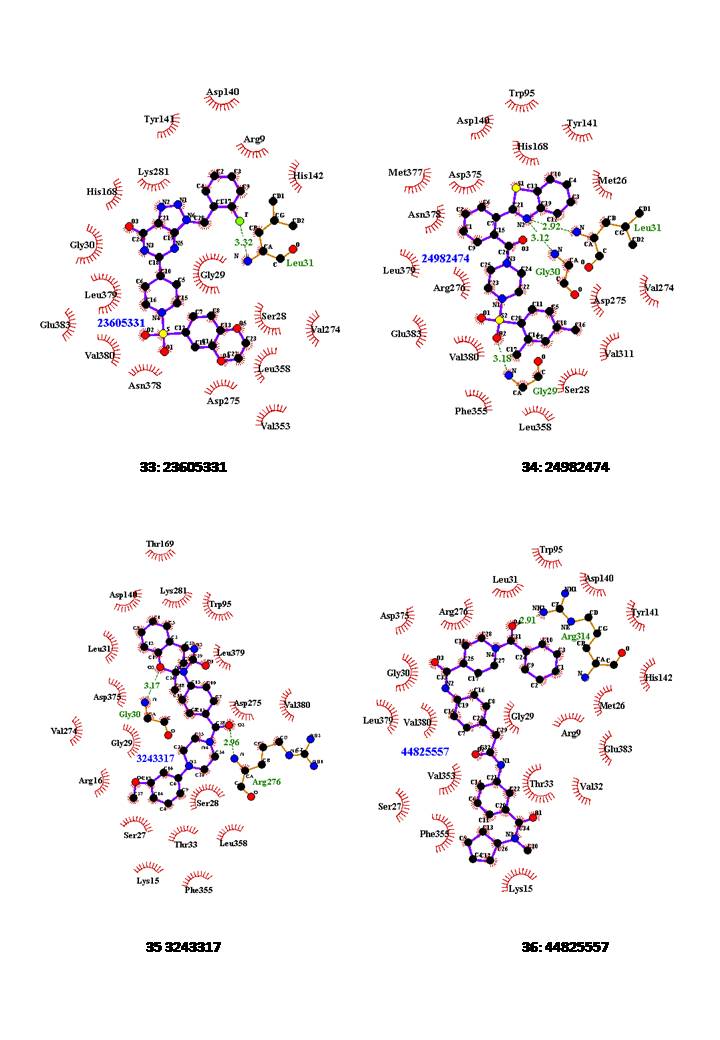

Supplement: Supplementary file 4 — Additional file 4: Figure S3: The top 45 compounds with the strongest binding confirmations are listed here, and the 2D interactions with Tps1 were also presented, which were prepared by Ligplot. (ZIP 920 KB) [file 40064_2013_782_MOESM4_ESM.zip › 8735656271096913_add3/8735656271096913_add11.jpeg]

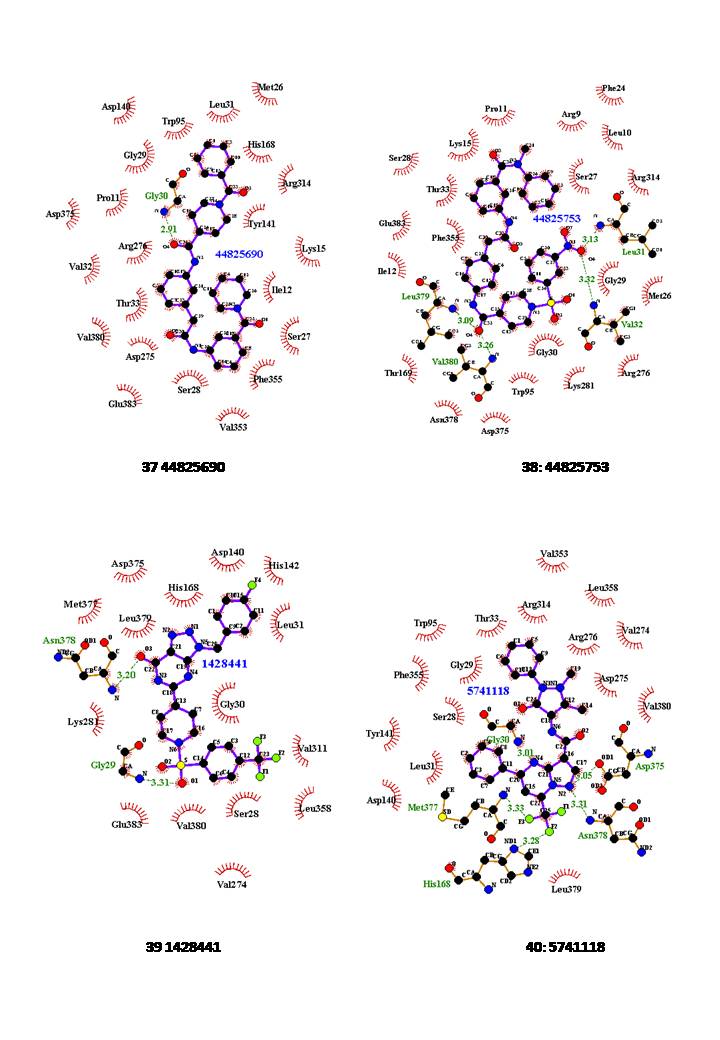

Supplement: Supplementary file 4 — Additional file 4: Figure S3: The top 45 compounds with the strongest binding confirmations are listed here, and the 2D interactions with Tps1 were also presented, which were prepared by Ligplot. (ZIP 920 KB) [file 40064_2013_782_MOESM4_ESM.zip › 8735656271096913_add3/8735656271096913_add12.jpeg]

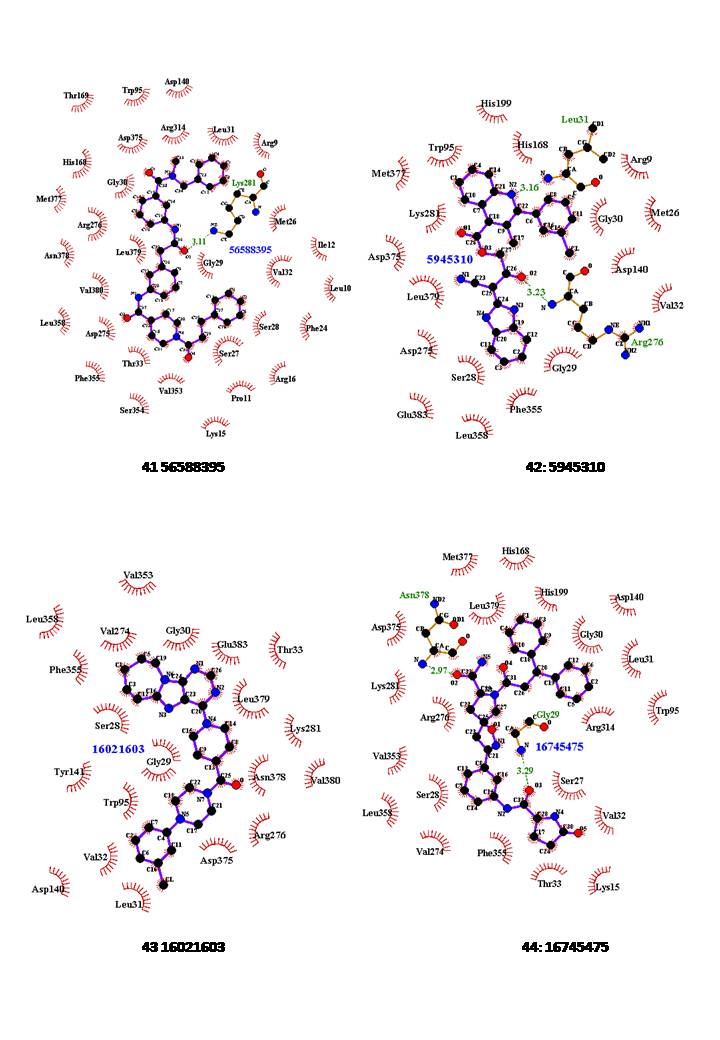

Supplement: Supplementary file 4 — Additional file 4: Figure S3: The top 45 compounds with the strongest binding confirmations are listed here, and the 2D interactions with Tps1 were also presented, which were prepared by Ligplot. (ZIP 920 KB) [file 40064_2013_782_MOESM4_ESM.zip › 8735656271096913_add3/8735656271096913_add13.jpeg]

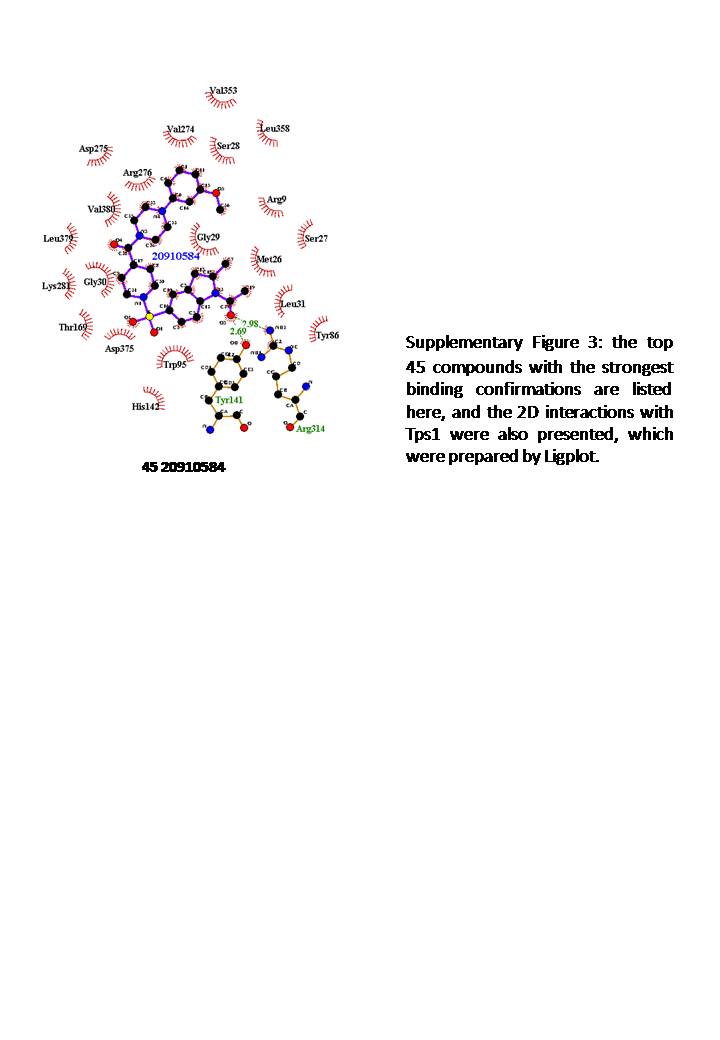

Supplement: Supplementary file 4 — Additional file 4: Figure S3: The top 45 compounds with the strongest binding confirmations are listed here, and the 2D interactions with Tps1 were also presented, which were prepared by Ligplot. (ZIP 920 KB) [file 40064_2013_782_MOESM4_ESM.zip › 8735656271096913_add3/8735656271096913_add14.jpeg]

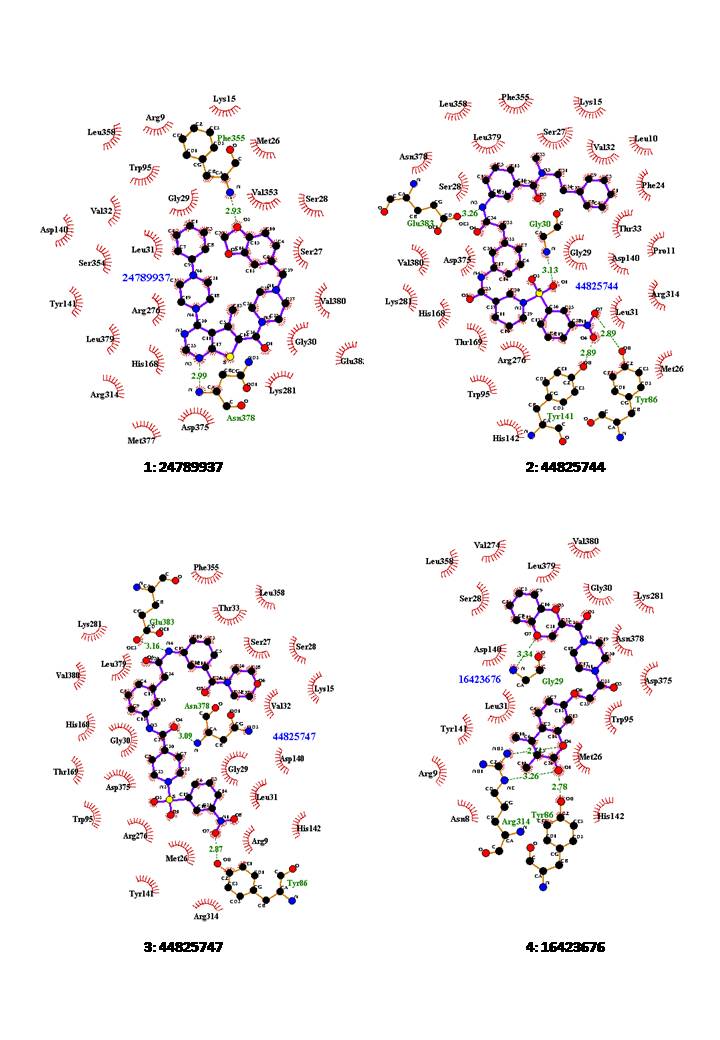

Supplement: Supplementary file 4 — Additional file 4: Figure S3: The top 45 compounds with the strongest binding confirmations are listed here, and the 2D interactions with Tps1 were also presented, which were prepared by Ligplot. (ZIP 920 KB) [file 40064_2013_782_MOESM4_ESM.zip › 8735656271096913_add3/8735656271096913_add3.jpeg]

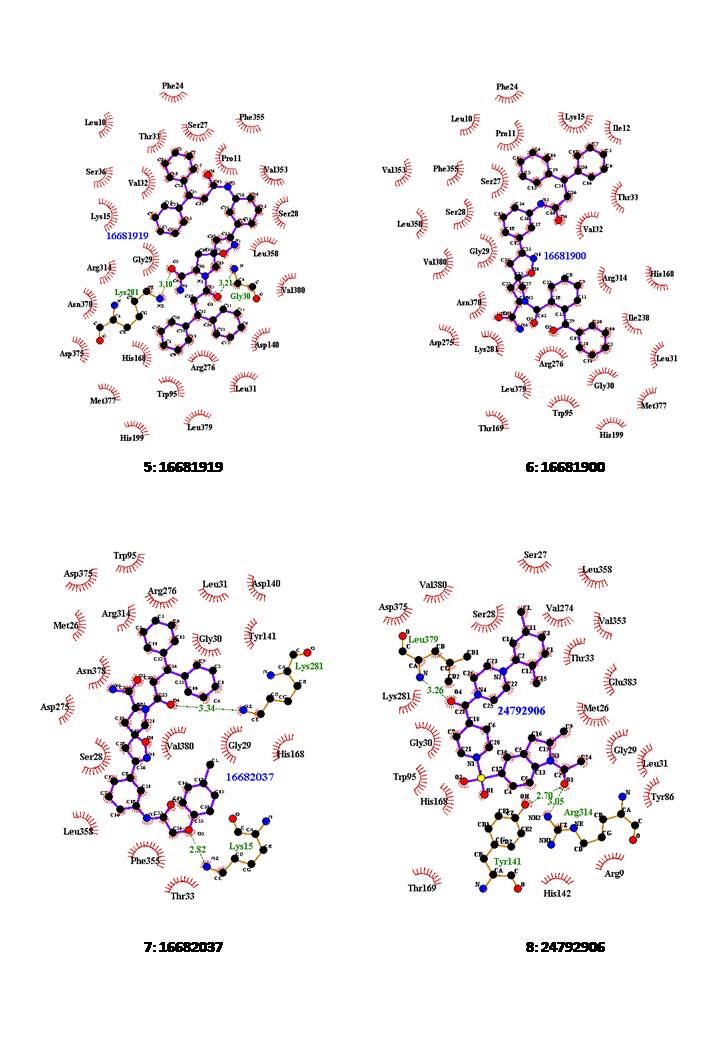

Supplement: Supplementary file 4 — Additional file 4: Figure S3: The top 45 compounds with the strongest binding confirmations are listed here, and the 2D interactions with Tps1 were also presented, which were prepared by Ligplot. (ZIP 920 KB) [file 40064_2013_782_MOESM4_ESM.zip › 8735656271096913_add3/8735656271096913_add4.jpeg]

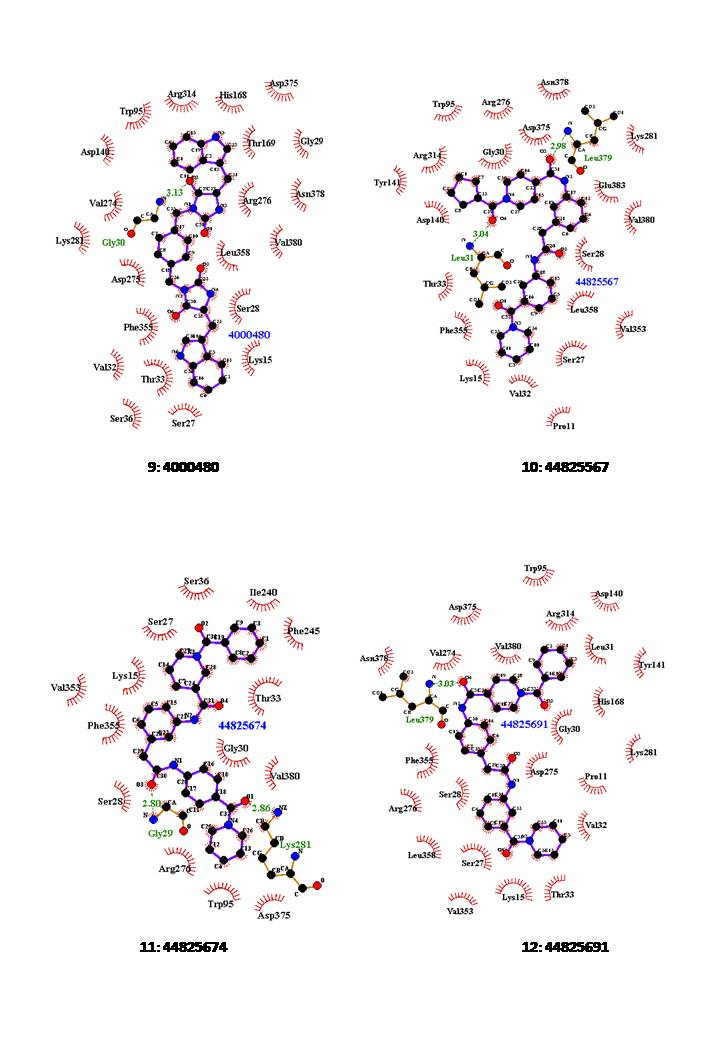

Supplement: Supplementary file 4 — Additional file 4: Figure S3: The top 45 compounds with the strongest binding confirmations are listed here, and the 2D interactions with Tps1 were also presented, which were prepared by Ligplot. (ZIP 920 KB) [file 40064_2013_782_MOESM4_ESM.zip › 8735656271096913_add3/8735656271096913_add5.jpeg]

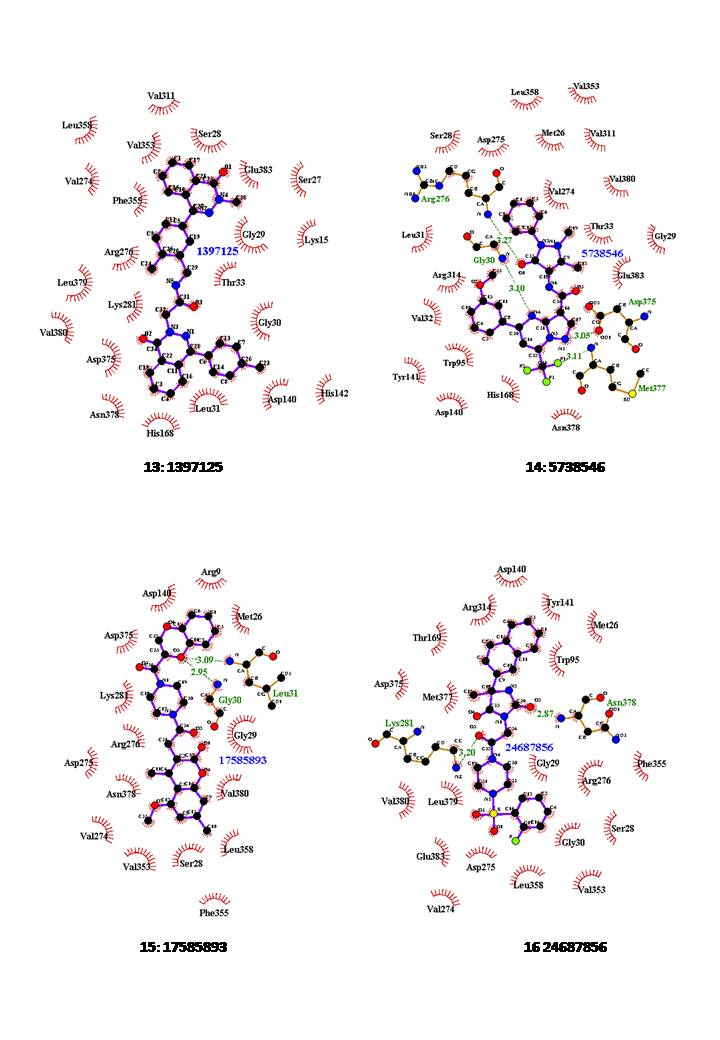

Supplement: Supplementary file 4 — Additional file 4: Figure S3: The top 45 compounds with the strongest binding confirmations are listed here, and the 2D interactions with Tps1 were also presented, which were prepared by Ligplot. (ZIP 920 KB) [file 40064_2013_782_MOESM4_ESM.zip › 8735656271096913_add3/8735656271096913_add6.jpeg]

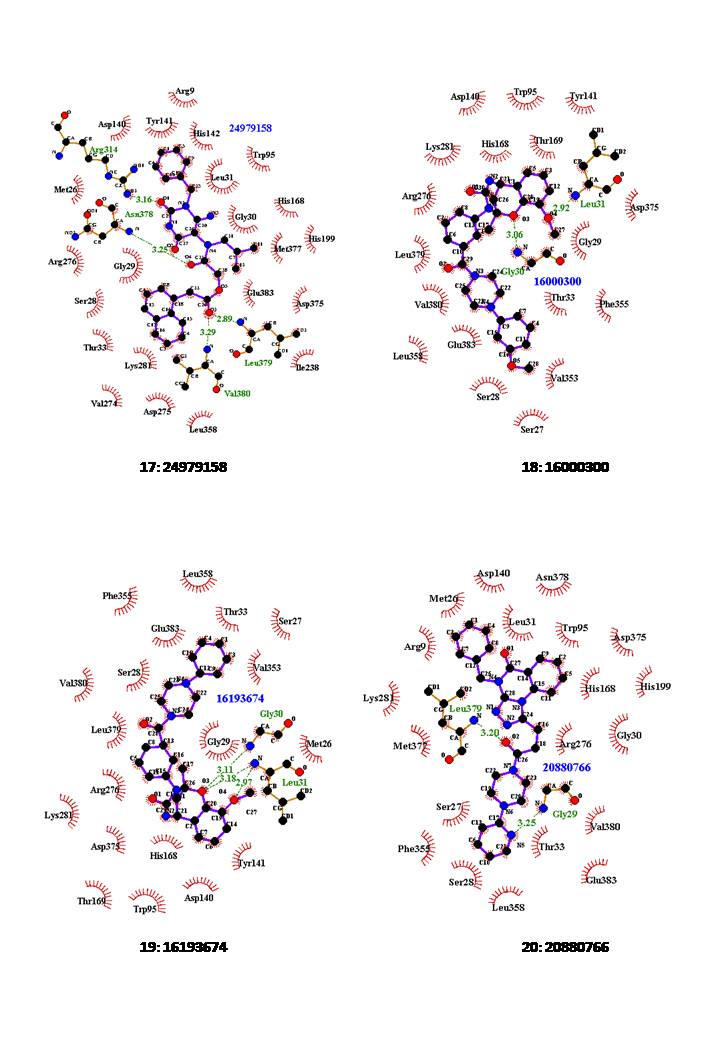

Supplement: Supplementary file 4 — Additional file 4: Figure S3: The top 45 compounds with the strongest binding confirmations are listed here, and the 2D interactions with Tps1 were also presented, which were prepared by Ligplot. (ZIP 920 KB) [file 40064_2013_782_MOESM4_ESM.zip › 8735656271096913_add3/8735656271096913_add7.jpeg]

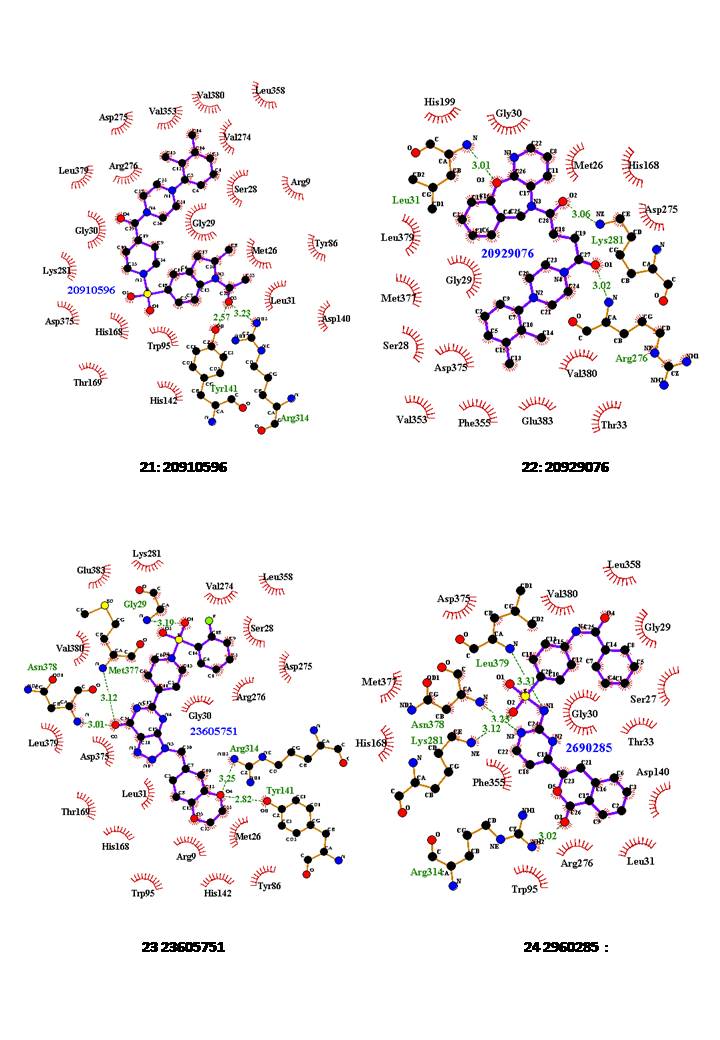

Supplement: Supplementary file 4 — Additional file 4: Figure S3: The top 45 compounds with the strongest binding confirmations are listed here, and the 2D interactions with Tps1 were also presented, which were prepared by Ligplot. (ZIP 920 KB) [file 40064_2013_782_MOESM4_ESM.zip › 8735656271096913_add3/8735656271096913_add8.jpeg]

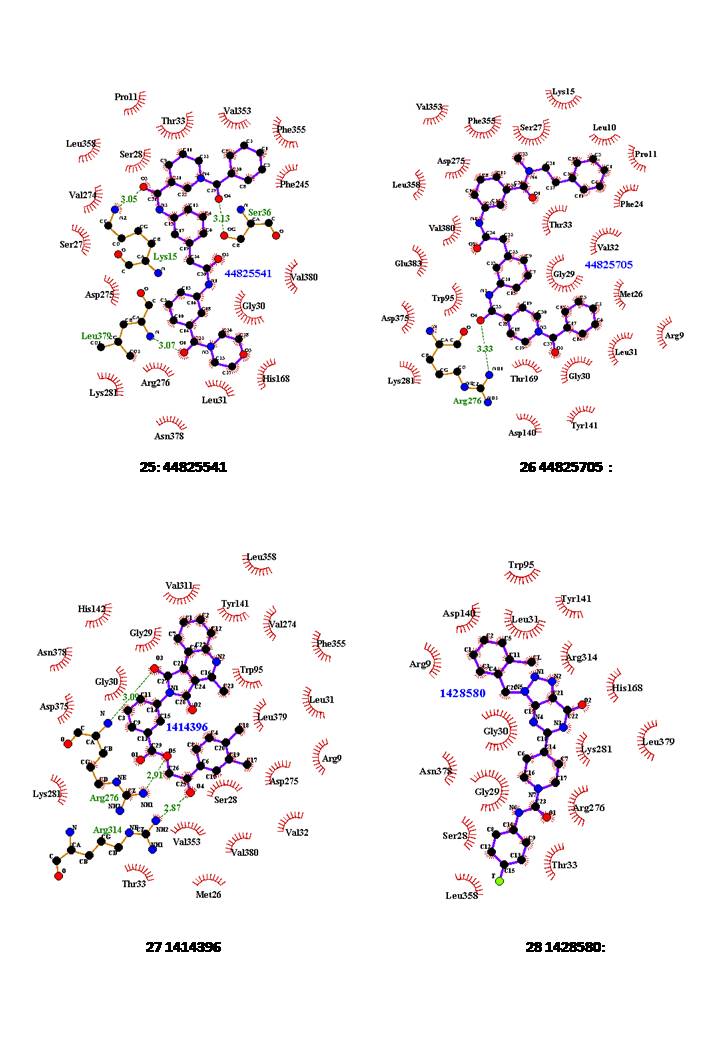

Supplement: Supplementary file 4 — Additional file 4: Figure S3: The top 45 compounds with the strongest binding confirmations are listed here, and the 2D interactions with Tps1 were also presented, which were prepared by Ligplot. (ZIP 920 KB) [file 40064_2013_782_MOESM4_ESM.zip › 8735656271096913_add3/8735656271096913_add9.jpeg]

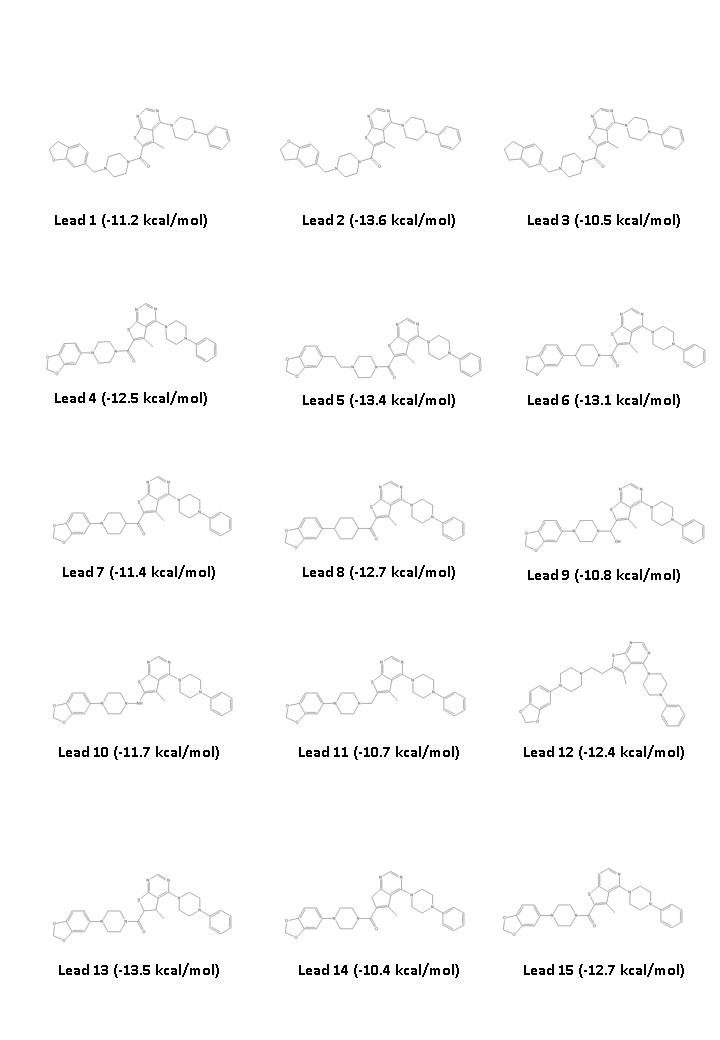

Supplement: Supplementary file 5 — Additional file 5: Figure S4: the 2D structures of the 26 modified compounds based on Compound 24789937 as the template, with the corresponding affinities were also included in the brackets. (ZIP 140 KB) [file 40064_2013_782_MOESM5_ESM.zip › 8735656271096913_add5/additional file 5_figure s4_a.JPG]

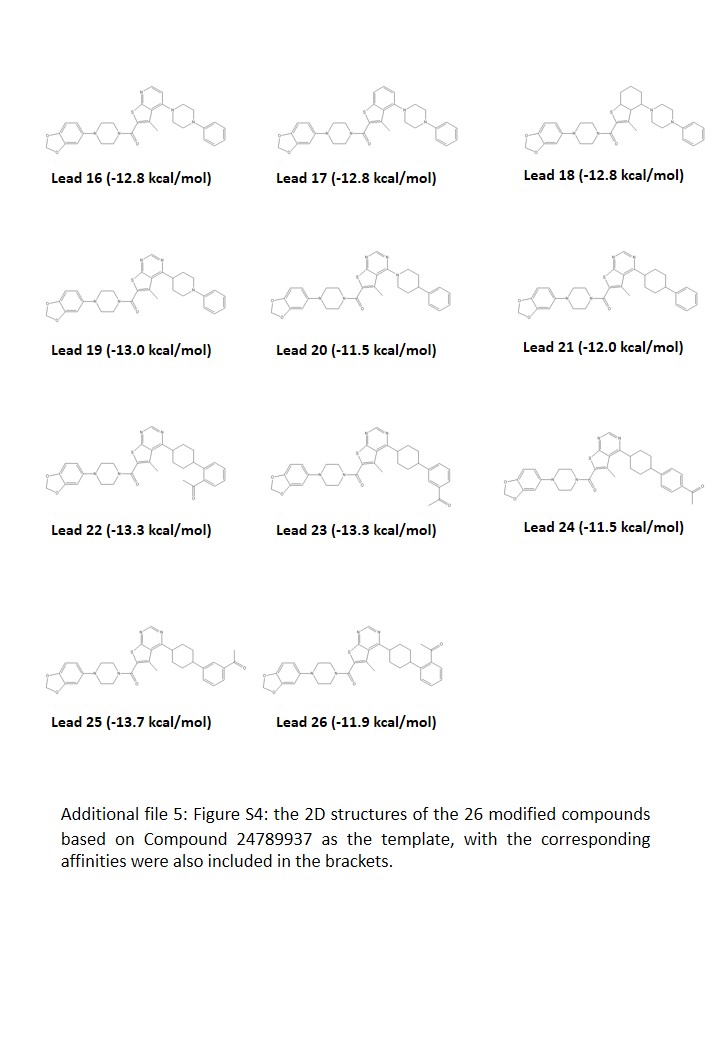

Supplement: Supplementary file 5 — Additional file 5: Figure S4: the 2D structures of the 26 modified compounds based on Compound 24789937 as the template, with the corresponding affinities were also included in the brackets. (ZIP 140 KB) [file 40064_2013_782_MOESM5_ESM.zip › 8735656271096913_add5/additional file 5_figure s4_b.jpg]
